# Nutrient Management Programs, Nitrogen Fertilizer Practices, and Groundwater Quality in Nebraska’s Central Platte Valley (U.S.), 1989–1998

**DOI:** 10.1100/tsw.2001.365

**Published:** 2001-11-21

**Authors:** Stan Daberkow, Harold Taylor, Noel Gollehon, Milt Moravek

**Affiliations:** Resource Economics Division, U.S. Dept. of Agriculture, Washington, DC 20036, USA

**Keywords:** nitrogen management programs, groundwater quality, nitrate, nitrogen fertilizer practices, Nebraska Central Platte Valley

## Abstract

Given the societal concern about groundwater pollution from agricultural sources, public programs have been proposed or implemented to change farmer behavior with respect to nutrient use and management. However, few of these programs designed to change farmer behavior have been evaluated due to the lack of detailed data over an appropriate time frame. The Central Platte Natural Resources District (CPNRD) in Nebraska has identified an intensively cultivated, irrigated area with average groundwater nitrate-nitrogen (N) levels about double the EPA"s safe drinking water standard. The CPNRD implemented a joint education and regulatory N management program in the mid-1980s to reduce groundwater N. This analysis reports N use and management, yield, and groundwater nitrate trends in the CPNRD for nearly 3000 continuous-corn fields from 1989 to 1998, where producers faced limits on the timing of N fertilizer application but no limits on amounts. Groundwater nitrate levels showed modest improvement over the 10 years of this analysis, falling from the 1989-1993 average of 18.9 to 18.1 mg/l during 1994-1998. The availability of N in excess of crop needs was clearly documented by the CPNRD data and was related to optimistic yield goals, irrigation water use above expected levels, and lack of adherence to commercial fertilizer application guidelines. Over the 10-year period of this analysis, producers reported harvesting an annual average of 9729 kg/ha, 1569 kg/ha (14%) below the average yield goal. During 1989-1998, producers reported annually applying an average of 162.5 kg/ha of commercial N fertilizer, 15.7 kg/ha (10%) above the guideline level. Including the N contribution from irrigation water, the potential N contribution to the environment (total N available less estimated crop use) was estimated at 71.7 kg/ha. This is an estimate of the nitrates available for denitrification, volatilization, runoff, future soil N, and leaching to groundwater. On average, between 1989-1993 and 1994-1998, producers more closely followed CPNRD N fertilizer recommendations and increased their use of postemerge N applications--an indication of improved synchrony between N availability and crop uptake.

* The opinions and conclusions expressed here are those of the authors and do not represent the views of the U.S. Dept. of Agriculture or the Central Platte Natural Resources District.

## INTRODUCTION

Nitrate contamination of ground- and surface water has long been recognized as a risk to human health and the environment[1]. In response to these risks, the U.S. has enacted a number of policies to reduce water pollution, including the Clean Water Act, the Safe Drinking Water Act, the Wellhead Protection Program, the Source Water Assessment Program, and the Sole Source Aquifer Protection Program. While federal policies may set national standards for water quality (e.g., 10 mg/l for N in drinking water), states and local areas often have the responsibility to develop and implement water quality programs to meet those standards. Given the regional variability in natural resources and economic activity, state and local jurisdictions, rather than federal agencies, may be able to more efficiently design and implement programs to manage N pollution of water resources. This is particularly appropriate for areas where N pollution is from nonpoint sources such as agricultural crop production.

A variety of federal, state and local policies have been proposed to address nonpoint source pollution generated by the agricultural sector[2]. These programs include (1) educating producers about best management practices (BMPs) and encouraging their voluntary adoption; (2) economic incentives to change producer behavior either through taxes (e.g., fertilizer tax) or subsidies (e.g., cost-sharing of BMPs); and (3) regulations or standards such as limits on nitrate emissions or required use of specific production practices (e.g., required split N applications or a ban on fall N application). The most widely used policy tools have been education and voluntary adoption of BMPs, occasionally with a cost share component. However, several states, with the encouragement of the federal government, have established programs to address groundwater N problems. For those programs where a measure of environmental outcome is available, the impact from changes in producer behavior, with respect to nutrient management, on environmental quality can be inferred.

This paper has two broad objectives: (1) to illustrate how a local jurisdiction in an agricultural area responded to the federal drinking water standard by developing a N management program for farmers, and (2) to assess the impact of the N management program on water quality and on farmer behavior with respect to N use and management.

This study analyzes the outcome of a 10-year program in central Nebraska to monitor groundwater nitrate levels, establish N use regulations and guidelines, and educate farmers on the importance of following the guidelines. Producers in this area faced some limits on timing of fertilizer application but no limits on amounts. The study documents the 1989–1998 trends in expected and actual yields, N application, N BMP adoption, and nitrates in the soil and irrigation water for nearly 3000 corn fields in central Nebraska. The specific questions we address are:

1. Did farmers adopt more realistic yield goals and more closely follow N use guidelines?
2. How has groundwater quality changed over this period?
3. How much N above crop needs is available for potential transport to the environment and what is the relative contribution of different management practices to excess N?
4. What N BMPs have farmers adopted since the local groundwater management program was initiated?

Summarizing the trends in the data indicate how farmers may have changed their behavior with respect to nutrient management in response to the local education and regulatory program. The results of this combined education and regulatory effort will be instructive for other agricultural areas of the U.S. faced with similar water quality concerns.

## WATER QUALITY MANAGMENT IN THE CENTRAL PLATTE VALLEY

One of the nation's aquifers most vulnerable to nitrate contamination is located in the Central Platte Valley of Nebraska (Fig. 1).

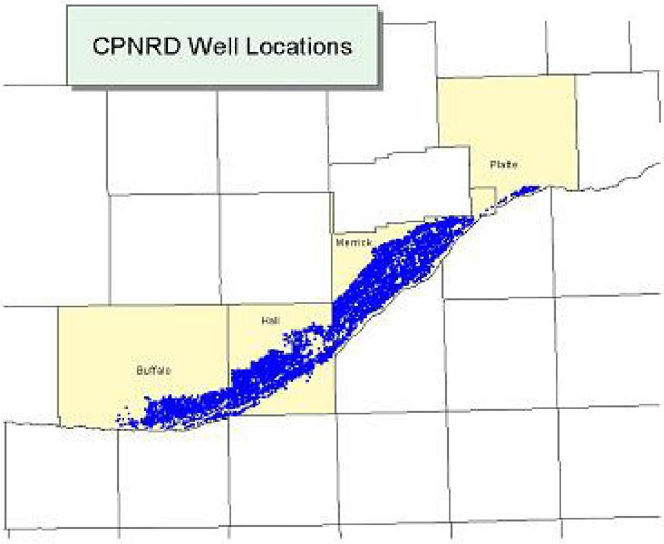

**FIGURE 1.** Location of Nebraska CPNRD continuous corn fields and irrigation wells used in this analysis.

A shallow, unconfined aquifer with overlying, highly permeable soils makes this area ideal for irrigated crop production and its aquifer susceptible to nonpoint nitrate contamination. The presence of large tracts of continuous corn production in this area results in intensive use of N fertilizer and irrigation water. Groundwater studies as early as the 1950s documented the presence of nitrates in this Platte River alluvial aquifer[3]. Succeeding studies noted the steady rise in groundwater N levels over the next 30 years to about double the U.S. Environmental Protection Agency's (EPA) safe drinking water standard of 10 mg/l (Fig. 2). A recent study found median nitrate levels of 25 mg/l in selected groundwater areas in the Central Platte Valley[4]. Furthermore, the underlying aquifer is connected hydraulically to the Platte River; this fact has water quality implications downstream from the Central Platte Valley[4]. The combination of a shallow water table, intensively irrigated agriculture, and permeable soils creates the potential for nitrate contamination of the aquifer.

The local multicounty agency responsible for addressing the growing concern about nitrate contamination of the region's groundwater is the Central Platte Natural Resources District (CPNRD)[6]. Beginning in the mid-1980s, the CPNRD promulgated a variety of field-level regulations and practices to improve N fertilizer management, including (1) soil and irrigation water tests to determine the quantity of N available for crop use, (2) the explicit recognition of an N-grain yield relationship for corn, (3) restrictions on the timing of commercial fertilizer applications, and (4) monitoring of irrigation water applications. The CPNRD guidelines combine field-level yield goals and a N fertilizer-yield relationship to compute estimates of total N need for each field[a]. From the estimates of total N need, the quantity of N provided by in-field sources (residual soil levels, irrigation water, and previous crop) is subtracted to determine the recommended level of N from commercial fertilizer[b]. The required soil test provides the residual soil N in 3 ft of soil while the required irrigation well tests provide the level of N from irrigation water. The CPNRD guidelines also attempt to improve fertilizer use efficiency by restricting commercial N applications in the fall and encouraging split and/or sidedress applications in the spring and early summer when N uptake by corn is most active. Finally, in recognition of the impact that irrigation water management can have on the leaching process, the CPNRD has instituted an irrigation water monitoring program.

## DATA

The data used for this analysis are from the annual (1989–1998) field-level reports that area farm operators are required to submit to the CPNRD. Data for each field includes: soil N test results, irrigation well water nitrate test results, N available from the previous crop, planned crop, yield goal, actual yield, commercial N fertilizer recommendations, actual N application levels, irrigation water application rates, inhibitor use, use of split applications, and use of a water meter. While the entire 10-year CPNRD database consists of over 50,000 field-level records, this analysis was restricted to only those fields growing corn for grain and for which at least 7 years of data were recorded during the 1989–1998 period. Hence, this analysis summarizes the annual production and nutrient management history of fields primarily planted to continuous corn.

## RESULTS

Given that the CPNRD collects field-level data on the major sources of N available for crop production as well as information needed for estimating N consumed by the crop, a modified N budget or mass-balance approach is used to summarize the data[8,9]. Schepers et al.[10] used a similar framework to analyze the 1988 CPNRD data. These approaches focus attention on N available in excess of crop use that is contributed to the air, water, or soil environment. We will define *excess N* as the estimated total amount of N available from all major sources less the estimated amount used by the crop (see appendix). This analy-

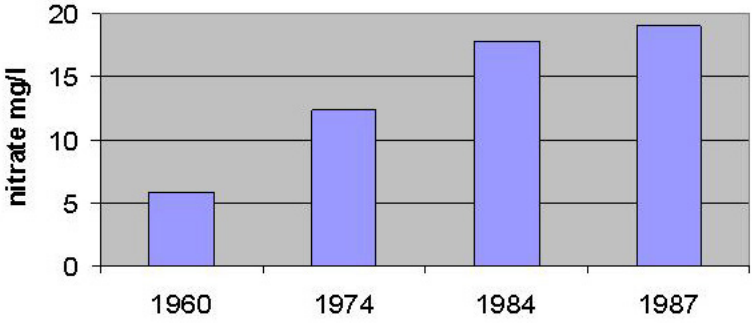

**FIGURE 2.** CPNRD groundwater nitrate levels for selected years[5].

[a] The actual N (lb/acre) fertilizer use function is 56 + 0.9Y/(1-0.0008 Y), where Y is the yield goal in bu/acre[7].

[b] In recognition of the uncertainties associated with the amounts of N needed and the availability of N from noncommercial sources, the CPNRD guidelines allow some flexibility. For those fields where the N soil residual is less than 56 kg/ha, the CPNRD guidelines require that a 56 kg/ha figure be used in the recommendation formula. In addition, regardless of the final recommendation formula, the CPNRD guideline suggests a 56 kg/ha minimum commercial N application. The 56-kg guideline minimum influences the recommended fertilizer levels only in fields with high residual soil carryover.

sis assumes that the functional form for the amount of N used by the crop is the same form as that used to estimate the amount of N needed by the crop[c].

## Yield Goals and Actual Yields

An integral part of the CPNRD N management program begins with producers' yield expectations. Yield expectations or goals are used to estimate the total amount of N needed to accommodate those yields. Yield goals are typically based on actual yields from the previous 5 years plus 5%. Producers in the CPNRD reported yields goals of about 11,298 kg/ha over the 1989–98 period, with the average increasing slightly from 11,235 in 1989–1993 to 11,360 kg/ha in 1994–1998 (Table 1)[d]. Actual yields were also higher in the second 5-year period (i.e., 9917 vs. 9540 kg/ha) but average yields were reduced in both periods due to adverse weather in 1993 and 1995. With the exception of 1993

**TABLE 1**
**Selected CPNRD Corn Production and Nitrogen Management Data, 1989–1998**

| | Units | 1989–1998 Average | 1989–1993 Average | 1994–1998 Average | Change 1989–1993 to 1994–1998 |
|---|---|---|---|---|---|
| **Number of corn fields** | Number | 2982 | 2893 | 3071 | NA |
| **Yield** | | | | | |
| Goal | kg/ha | 11,323 | 11,266 | 11,386 | 120 |
| Actual | kg/ha | 9754 | 9565 | 9929 | 364 |
| Deviation | kg/ha | 1569 | 1701 | 1457 | –244 |
| **N application** | | | | | |
| Actual | kg/ha | 162.6 | 158.7 | 166.4 | 7.7 |
| CPNRD recommendation | kg/ha | 146.7 | 137.1 | 155.8 | 18.7 |
| Deviation | kg/ha | 15.9 | 21.6 | 10.6 | –11.0 |
| **Irrigation water** | | | | | |
| Nitrate levels | Mg/l | 18.5 | 18.9 | 18.1 | –0.8 |
| N from 22.9 cm water | kg/ha | 41.5 | 42.4 | 40.7 | –1.7 |
| Actual application | cm | 31.8 | 31.5 | 32.3 | 0.8 |
| N from actual water application | kg/ha | 58.7 | 58.7 | 58.7 | 0.0 |
| **Residual soil N** | kg/ha | 92.5 | 202.9 | 82.6 | –20.3 |
| **Total N available** | kg/ha | 314.4 | 321.1 | 308.1 | –13.0 |
| **N used by crop** | kg/ha | 242.9 | 239.1 | 246.5 | 7.4 |
| **Excess N total** | kg/ha | 71.5 | 82.0 | 61.6 | –20.4 |
| Source: | | | | | |
| High yield goal | kg/ha | 33.1 | 35.6 | 30.7 | –4.9 |
| Water in excess of 22.9 cm | kg/ha | 17.1 | 16.3 | 18.0 | 1.7 |
| Remainder | kg/ha | 21.3 | 30.1 | 12.9 | –17.2 |
| N applied in excess of N recommended | kg/ha | 15.9 | 21.6 | 10.5 | –11.1 |
| Remainder due to 56 kg/ha minimum | kg/ha | 5.4 | 8.5 | 2.4 | –6.1 |
| **N management** | | | | | |
| Share of fields with: | | | | | |
| Inhibitor (1989–98) | % | 22.3 | 22.7 | 21.9 | –0.8 |
| Fall application (1990–98) | % | NA | 3.0 | 0.8 | –2.2 |
| Split application (pre and post) (1989–98) | % | 65.8 | 68.7 | 63.1 | –5.6 |
| Sidedress application (1994–98) | % | NA | NA | 75.8 | NA |
| Water meter (1991–98) | % | NA | NA | 19.5 | NA |
| Share of total N applied side-dress (1993–98) | % | NA | NA | 52.0 | NA |

NA = not applicable/not available.

[c] Various functional forms were explored to estimate N uptake by the crop. The CPNRD instructions to producers suggest that a fixed coefficient of 2.3 kg of N/100 kg of expected (or actual) yield should be used to estimate the amount of N needed (or consumed). The most current Nebraska Extension recommendation suggests the following relationship: N needed (lb/acre) = 35 + 1.2*yield goal, where yield goal is in bu/acre[11]. This analysis used the relationship reported in Supalla et al.[7] and footnote a to estimate both N needed and N used.

[d] Since the entire universe of CPNRD farmers who grew continuous corn is included in the analysis, no statistical test of the differences in means over time was conducted.

and 1995, actual yields were fairly homogenous across the CPNRD: over 65% of the fields reported actual yields of between 9415 and 12,553 kg/ha, and only about 10% of the fields reported less than 6277 kg/ha or more than 12,553 kg/ha.

Average yield goals exceeded actual yields by considerably more than 5% in all years. On average, yield goals exceeded actual yields by more than 1569 kg/ha (i.e., over 15%) over the 1989–1998 period. While the deviation between actual and expected yields declined from about 1695 to 1444 kg/ha from the first 5-year period to the last, much of the change was due to the severe actual yield decline in 1993. Excluding 1993 data from the 1989–1993 period shows that deviations between expected and actual yields actually increased — an indication that farmers in the CPNRD are not adjusting their yield goals to reflect historic levels[e].

Several producers reported that their yield goals were much greater than actual yields. For example, during the 1989–1998 period, about 17% of the fields had yield goals exceeding actual yields by more than 3138 kg/ha and another 20% reported expected yields exceeding actual yields by 1883 to 3138 kg/ha. Only about 24% of the fields reported that actual yields were within +628 kg/ha of expected yields. Another 6% of the fields had actual yields of 628 kg/ha or more above the expected yields. Hence, on average, only about 30% of the fields were operated by producers who either (1) closely matched (within 5%) yield goals with actual yields or (2) exceeded yield goals during 1989–1998.

## N Recommended and N Applied

Producers in the CPNRD have exhibited a chronic tendency to apply N fertilizer in excess of the CPNRD recommended rate. The CPNRD recommended N fertilizer rate is based on the amount of N required to meet the expected yield minus the amount of N in the soil, in the irrigation water, and from a previous legume crop. The CPNRD minimum N recommendation is 56 kg/ha, which was recommended for about 9% of the fields during 1989–1998. From the 1989–1993 to the 1994–1998 periods, the CPNRD average recommendation increased from over 136.7 kg/ha of N to 155.8 kg/ha, partly due to higher expected yields, but primarily due to lower soil N levels. The levels of applied N fertilizer rose slightly, from about 159.2 to 165.9 kg/ha of N between the same two time periods. Over the 10 years of this analysis, producers applied about 15.7 kg, or about 10%, more N than was recommended, but the deviation appears to be declining. During 1989–1993, the amount of N that producers applied was nearly 22.4 kg/ha above the recommended rate, but the deviation declined to about 10.1 kg in 1994–1998. However, some fields were treated with N fertilizer far in excess of the recommended levels. For example, between 1989 and 1998 over 9% of the fields received more than 84.1 kg/ha of N above the recommended rates. More encouraging is the finding that nearly half of the fields during that time received applications that were within +28 kg/ha of the recommended rate. In addition, over 13% of the fields received 28 kg/ha of N or more below the CPNRD recommendation.

## N in Irrigation Water

Annual test results for nitrates in irrigation water used in the CPNRD serve two purposes: they measure (1) groundwater quality over time and (2) the level of N available in irrigation water for corn production (when combined with the annual amount of irrigation water applied)[f]. Nitrate levels within the CPNRD averaged over 18 mg/l during the 10-year period of the study, which is well above the EPA's N standard for drinking water. However, there is considerable variation in nitrate levels throughout the region. About 21% of the fields were serviced by wells with less than 10 mg/l nitrate levels, but 2 to 3% of the fields reported nitrate mg/l levels greater than 40.

There was a modest improvement in the average nitrate levels from 18.9 to 18.1 mg/l from the first to second 5-year period. The amount of N available from 22.9 cm of irrigation water declined accordingly[g]. However, farmers reported using about 31.8 cm of irrigation water annually, which results in about 58.3 kg/ha of N, on average, available to the crop through irrigation[h]. A significant number of producers applied irrigation water in excess of the expected 22.9 cm during 1989–1998. Over 15% of the fields reported applying more than 48.3 cm of irrigation water annually — an indication that some producers are in need of an irrigation management program in conjunction with nutrient management assistance.

## Soil N

In addition to N fertilizer and nitrates in the irrigation water, residual soil N is available for plant nutrition. The CPNRD preseason required soil test is an estimate of soil stored N. Average residual soil N has shown a substantial decline, from about 103.1 kg/ha of N available to nearly 82.9 kg/ha between 1989–1993 and 1994–1998 — an indication that farmers are beginning to more fully utilize this source of N as well as better manage N

[e] The 1989–1992 data indicate that the deviation between actual and expected yield was about 1193 kg/ha compared to the 1994–1998 average of about 444 kg/ha. Actual yields were also lower than the long-term average in 1995 and excluding that year from the 1994–1998 average results in an average deviation of about 1161 kg/ha — similar to the 1989–1992 average.

[f] Zlotnik et al.[12] have shown that, with proper protocols, irrigation wells are a reliable source of information about the areal extent of nonpoint source contamination of groundwater.

[g] The N in 22.9 cm of irrigation water is the amount of N-credit for irrigation water that the CPNRD includes in its fertilizer recommendation. Note that in 1993, irrigation water use was very low, which significantly reduced average water use for the 1989–1993 period. A reviewer pointed out that 1993 was an extremely wet year, which resulted in a significant aquifer recharge and may have contributed to the decline in nitrate concentration after 1993.

[h] According to the CPNRD staff, producer estimates of annual water use are very crude and likely very conservative, since few producers had water meters prior to 1996. Because of the critical role that irrigation water plays in N leaching, the CPNRD required the use of water meters after 1996.

applications to reduce end-of-season residual[i]. As with many of the indicators reported by the CPNRD, there is considerable variation in residual soil N. For example, over the 10-year period, about 10% of the fields reported more than 168.1 kg/ha of available soil N, although the share of fields in this category has declined dramatically. At the other extreme, the number of fields reporting exactly 56 kg/ha of available N is increasing as residual soil N levels fall. The CPNRD specifies that any soil test reporting less than 56 kg/ha of N is to be set equal to 56 in the formula for determining the N fertilizer recommendation, which slightly lowers the average N fertilizer recommendation.

## N Available and N Used

Even though a precise estimate of the total N available for crop production and the total used by the crop is not possible, the CPNRD data can be used to approximate the relative changes in these two quantities (and their difference) over time. The difference between total N available and N used is an estimate of N available for release into the environment. Total available N (i.e., the sum of fertilizer applied, nitrates in irrigation water and the soil — N from previous crops was negligible since these are primarily continuous corn fields) declined about 12.3 kg/ha over the 1989–1998 period due to the decline in soil nitrates and modest decline in irrigation water nitrate levels.

N used by the crop is directly related to actual yields and showed an increase between the 1989–1993 and 1994–1998 periods. However, the low yields in 1993 influenced the average for the earlier period[j]. The difference between N available and used fell over 20.2 kg/ha between the two 5-year periods, indicating that less N was available for escape into the environment, especially into groundwater. As with the other N management indicators, there was considerable variation in our estimates of excess N. During 1989–1998, N on about 29% of the fields was managed in a manner that left little unused by the corn crop, while at the other extreme there was another nearly 15% of the fields that left over 140.1 kg/ha of N at risk of either leaching to groundwater, remaining in the soil, running off in surface water, or being lost from denitrification or volatilization.

Given the CPNRD data and framework for recommending N fertilizer application rates, it is possible to allocate the relative sources of excess N among four factors: high yield goal, irrigation water applied in excess of 22.9 cm, N fertilizer applied above recommended levels, and the 56 kg/ha minimum N recommendation rule (see Appendix). For the 1994–1998 period, about 30.3 kg/ha excess N is attributable to a high yield goal, 17.9 kg/ha for water use in excess of 22.9 cm, 10.1 kg/ha for fertilizer application greater than recommended, and about 2.2 kg/ha due to the 56-kg minimum rule. With the exception of irrigation water, which increased slightly between 1989–1993 and 1994–1998, all of the sources of excess N declined[k]. The 56-kg minimum rule is less of a contributing factor because of the decline in residual soil nitrate. One of the most significant behavioral changes between 1989–1993 and 1994–1998 was that producers reported less deviation from the CPNRD recommended fertilizer application rate.[l]

## N Management

The CPNRD also encourages producers to minimize the potential for nitrate leaching by adopting fertilizer BMPs. The most common recommended practices involve the use of inhibitors, improved fertilizer application timing, and enhanced irrigation water management. N inhibitors were used on about 20% of the fields with little change in the rate over the 1989–1998 period. Fall fertilizer application has been discouraged or banned in most of the region, which is reflected in the data: only about 3% of the fields reported fall fertilization during 1989–1993 and that figure dropped to less than 1% during 1994–1998. However, fields using split (pre- and postemergence) fertilizer application declined from about 68% of the fields to about 63% between the two 5-year time periods. While fertilizer sidedress (i.e., postemerge application) quantity data is not available for 1989–1993, the annual data for 1994–1998 show a significant increase in both the number of fields receiving N as a sidedress treatment and the share of total N applied sidedress. Because of the close linkage between irrigation management and N leaching, the CPNRD has now required producers to add a water meter to their wells, which provides producers with information about the quantity of water used. The use of water meters, however, was modest (i.e., less than 20% of the fields).

## CONCLUSIONS

Agricultural areas faced with groundwater N levels exceeding the EPA's drinking water standard will likely continue to be encouraged to develop and implement local programs to address N fertilizer use and management. The N management program implemented in central Nebraska illustrates the type of information program managers will need in order to document improvements in water quality as well as impacts on farmers' yields and N fertilizer practices. Also, the type of georeferenced, field-level information required from farmers in the CPNRD is an example of the data needed by program managers to efficiently target their enforcement resources. Furthermore, the CPNRD framework may be used by federal or state environmental agencies to assess the strengths and weaknesses of nutrient management programs in other areas of the U.S.

The results from this analysis are congruent with the conclusions of other studies of producer behavior with respect to N use and management. Within the CPNRD, available N exceeded N

[i] A reviewer pointed out that silt-loam soils can contribute an additional 80 to 100 kg/ha of N from mineralization during the growing season.

[j] Without 1993, N used by the crop averaged 248.6 kg/ha, compared to an average of 239.1 kg/ha with 1993 data.

[k] Due to annual variation in rainfall, farmers may apply irrigation water in excess of 22.9 cm in a given year. Irrigation water in excess of consumptive use can contribute to N leaching. We did not estimate consumptive irrigation water use in this analysis, but we note that N in irrigation water applied in excess of 22.9 cm is not fully accounted for in the N recommendation.

[l] Note that some of the improvement between the two 5-year periods in these excess N categories is due to the relative yield declines of 1993 compared to 1995.

crop use, which is similar to findings in other studies of agricultural areas[9]. Much of this excess N in the CPNRD was due to farmers not only applying N fertilizer above crop requirements but above CPNRD recommended levels. We estimated that on average during 1989–1998, about 45% of the excess N is attributable to an optimistic yield goal by producers, 25% due to irrigation water application above the expected annual consumptive use (i.e., 22.9 cm), and the remaining 30% due to failure to follow the CPNRD guidelines. Given the critical role that yield goals play in N recommendations in Nebraska, as well as in many other corn producing states, N management programs may need to encourage producers to form more realistic yield expectations or make N recommendations based on actual yields. Furthermore, this study showed that producer behavior with respect to forming realistic yield goals or closely following N recommendations changed rather slowly in response to a N education program. In addition, other studies have documented that farmers often apply N in excess of both the recommended rate and the profit-maximizing rate[13]. Babcock and Blackmer[14] have shown that risk-neutral farmers have incentives to apply more N fertilizer than is needed in order to reduce the probability of low yields.

During 1994–1998, producers increased their use of postemerge N fertilizer applications in terms of both the share of fields and share of N fertilizer applied as sidedress. The adoption of more intensive postemerge fertilizer applications, which improve the synchrony between N availability and crop uptake, may be an indication that producers found this particular practice economically rewarding. Profitability is often cited as one of the critical factors necessary to facilitate voluntary BMP adoption[2]. Conversely, as Bosch and Pease[15] point out, uncertainty and producer risk aversion may have slowed the adoption of other BMPs, such as nitrification inhibitors.

Finally, the results indicate that modest changes in N use and management result in modest changes in groundwater quality, which is the general conclusion of a number of economic studies[16]. Compared to the previous 30 years, from 1960 to 1987, where groundwater nitrate levels rose from about 5 mg/l in 1960 to about 19 mg/l in the late 1980s, the CPNRD program appears to have at least slowed the trend toward increased nitrate concentration in the region's groundwater. However, nearly 80% of the fields continue to report use of irrigation water with nitrate levels in excess of the EPA's standard for drinking water. More aggressive programs may be necessary to produce nonmarginal changes in groundwater quality in the longer run, especially regarding irrigation water management.

## ACKNOWLEDGMENTS

The authors want to acknowledge the valuable assistance and cooperation of Sandy Noecker of the CPNRD. Tommy Dalgaard, Dennis Francis, Jan Poulisse, and Roy Spalding made helpful comments on earlier drafts of this paper.

| | | |
|---|---|---|
| Received: | July | 18, 2001 |
| Revised: | November | 6, 2001 |
| Accepted: | November | 7, 2001 |
| Published: | November | 21, 2001 |

## APPENDIX
## Algebraic Derivation Used to Estimate the Sources of Excess Nitrogen

$$\text{EXCESSN} = \text{TOTN} - \text{NUSE} \tag{1}$$

$$\text{TOTN} = \text{APPN} + \text{SOILN} + \text{WATN} + \text{PREVN} \tag{2}$$

$$\text{NREC} = \text{NEEDN} - [\text{SOILN} + 9\text{WATN} + \text{PREVN}] \tag{3}$$

If (NEEDN – [SOILN + 9WATN + PREVN]) is <56, then NREC = 56

where

EXCESSN = the estimate of annual N available in excess of crop use,
TOTN = the estimate of total annual N available for crop production,
NUSE = N used by the crop based on actual yield,
APPN = annual N fertilizer applied,
SOILN = residual soil N,
WATN = N available from all irrigation water,
9WATN = N available from 22.9 cm of irrigation water,
PREVN = N from previous crop,
NREC = recommended N fertilizer application rate,
NEEDN = N required to meet yield goal, and
R = N increase due to 56 kg/ha minimum recommendation rule (where R = 56 - (NEEDN - [SOILN + 9WATN + PREVN]) if (NEEDN – [SOILN + 9WATN + PREVN]) < 56, and R = 0 otherwise).

Substitute Eq. 2 into Eq. 1:

$$\text{APPN} + \text{SOILN} + \text{WANT} + \text{PREVN} - \text{NUSE} = \text{EXCESSN} \tag{4}$$

Solve Eq. 3 for SOILN, substitute into Eq. 4, and rearrange terms:

$$(\text{APPN} - \text{NREC}) + (\text{WATN} - 9\text{WATN}) + (\text{NEEDN} - \text{NUSE}) + R = \text{EXCESSN} \tag{5}$$

where $R = 0$ when $\text{NREC} \geq 56$ and $R > 0$ when $(\text{NEEDN} - [\text{SOILN} + 9\text{WATN} + \text{PREVN}]) < 56$

The first term is the difference between N fertilizer applied and the CPNRD recommendation; the second term is the amount of N in irrigation water in excess of 22.9 cm; the third term is the difference between N needed to meet the yield goal and the N used based on actual yield; and the last term is the remainder due to the 56 kg/ha minimum N recommendation.
